# What is the impact of daily oral supplementation of vitamin D3 (cholecalciferol) plus calcium on the incidence of hip fracture in older people? A systematic review and meta‐analysis

**DOI:** 10.1111/opn.12492

**Published:** 2022-07-17

**Authors:** Preethy Manoj, Rosemarie Derwin, Sherly George

**Affiliations:** ^1^ School of Nursing and Midwifery The Royal College of Surgeons in Ireland (RCSI) University of Medicine and Health Sciences Dublin 2 Ireland; ^2^ Connolly Hospital, Blanchardstown Dublin 15 Ireland

**Keywords:** hip fracture, older adults, vitamin D3 and calcium

## Abstract

**Introduction:**

Hip fractures have a huge impact in reducing the quality of life and increasing mortality. This review aims to assess the impact of daily oral supplementation of vitamin D3 plus calcium on the incidence of hip fracture in people over 65 years.

**Methods:**

PRISMA guidelines were followed and RCTs that evaluated the effectiveness of daily oral supplementation of vitamin D3 plus calcium in preventing hip fracture in adults over 65 years were included in the study. The databases such as Cochrane Library, Embase, Medline, PubMed, CINAHL, Web of Science and Scopus were searched from October 2019‐ January 2020.The Cochrane risk of bias tool was used to check the quality of the included studies. A meta‐analysis with fixed effect model using Review Manager (Revman 5.3) was used to analyse the data.

**Results:**

The meta‐analysis of seven RCTs on vitamin D3 plus calcium supplementation and hip fracture (*n* = 12,620) identified odds ratio (OR) of 0.75; 95% Confidence interval (CI): 0.64, 0.87; *p* = .0003. Daily oral supplementation of 800 IU of Vitamin D3 plus 1200 mg of calcium was found more effective (*n* = 5676 participants; OR = 0.69; 95% CI: 0.58, 0.82; *p* < .0001) than daily oral supplementation of 800 IU of Vitamin D3 plus 1000 mg of calcium (*n* = 6555,OR = 1.08; 95% CI: 0.74, 1.56; *p* = .70) in reducing hip fracture. A meta‐analysis of the seven RCTs to identify the incidence of non‐vertebral fracture gave the OR of 0.80; 95% CI: 0.72, 0.89; *p* < .0001. A meta‐analysis of three RCTs on femoral neck bone mineral density (BMD) (*n* = 483) gave a mean difference of 1.21; 95% CI: ‐0.79, 3.20; *p* = .24.

**Conclusion:**

Daily oral supplementation 800 IU of vitamin D3 plus 1200 mg of calcium reduces hip fracture and non‐vertebral fracture in older people. Administering vitamin D3 and calcium supplements had no effect in increasing the femoral neck BMD.

**Implications for practice:**

Even though it is evident from the review that optimal daily intake of vitamin D3 plus calcium supplementation help in the prevention of fracture, it is only one essential element in fracture prevention. Also, people who are on dietary supplements should be compliant with same for better result. Efforts to prevent bone loss and osteoporosis should begin from an early age. It includes maintaining a healthy lifestyle, optimal intake of calcium and vitamin D3, proper nutrition, adequate exposure to sunlight, exercise etc. Proper education on healthy lifestyle, avoiding risk factors like smoking, caffeine, alcohol and awareness of bone health should continue throughout life with emphasis during menopause when increased bone loss is expected.

**What does this research add to existing knowledge in gerontology?**

- Meta‐analysis of the included studies showed remarkably favourable results in the vitamin D3 and calcium group in reducing the incidence of hip fracture and all non‐vertebral fractures.
- Administering 800 IU of cholecalciferol and 1200 mg of calcium daily is more effective in reducing hip fracture and non‐vertebral fracture than 800 IU of cholecalciferol and 1000 mg of calcium supplementation.
- Vitamin D3 and calcium supplementation showed no effect in increasing the femoral neck bone mineral density.

**What are the implications of this new knowledge for nursing care with older people?**

- Have effective strategies in place to administer 800 IU of cholecalciferol and 1200 mg of calcium daily to reduce the incidence of fracture in older people and thus improve their quality of life, independence and general well‐being.
- This strategy to reduce fractures in older people will result in economic benefits for both patients and healthcare systems.

**How could the findings be used to influence policy or practice or research or education?**

- The policy makers should acknowledge vitamin D3 plus calcium supplementation as one essential element in fracture prevention.
- To improve the quality of care of the older people, promoting positive ageing should be incorporated into the nursing curriculum.
- Awareness of bone health should continue throughout life with emphasis during menopause when increased bone loss is anticipated.
- Future research on approaches to prevent hip fracture in older people will help to improve their quality of living, decrease long‐term disability and dependence.

## 1 INTRODUCTION

Preventing fractures in older people is a priority as the ageing population is increasing and the incidence of fracture increases with age. Hip fractures pose highest demand on resources and have the greatest impact on patients due to high mortality, long‐term disability and loss of independence. Also, the individual involved might require long‐term care which will increase the healthcare expense (Gannon et al., 2007). Lin et al. (2018) pointed out that the mortality of the hip fracture patients in thirty days is 19%. It is estimated that the annual incidence of hip fracture worldwide will increase from 1.6 million in 2000 to at least 4.5 million in 2050 (Wiklund et al., 2016).

Yao et al. (2019) reported that approximately one in two women and one in five men over 50 years will experience an osteoporotic fracture in their remaining lifetime. According to Nowson (2010), osteoporosis is the biggest cause of fracture in older people. As bone loss happens without warning signs, osteoporosis is often considered a ‘silent disease’ which has been reported in people of all ethnic backgrounds (Sunyecz, 2008). It is described that a woman's risk of hip fracture is equivalent to her combined risk of breast, uterine and ovarian cancer (Sunyecz, 2008). Once the bone matrix is lost, there are no known therapeutic interventions to restore bone mass to normal. Calcium is the principal component of the bone matrix (Salovaara et al., 2010). Vitamin D regulates calcium and bone metabolism (Kröger et al., 2010).

Calcium and vitamin D supplements are inexpensive and generally well tolerated (Kessenich, 2007). There have been a few meta‐analyses that have assessed the benefits of vitamin D and calcium supplementation in preventing bone loss, fracture, falls, improving strength, physical performance, etc. (Bischoff‐Ferrari et al., 2007, 2009; Jackson et al., 2007; Latham et al., 2003; Lips et al., 2010; Wallace et al., 2015; Weaver et al., 2016). These reviews included calcium only studies, vitamin D only studies, combined vitamin D and calcium studies. The efficacy of daily oral supplementation of calcium plus vitamin D3 on the incidence of hip fracture and non‐vertebral fracture in men and women over 65 years of age (community dwelling and long‐term care [LTC] residents) with or without risk of fracture remain equivocal. The fracture risk associated with differences in concentrations of 25‐hydroxyvitamin D (25[OH]D) in observational studies and the risk of fracture associated with supplementation of vitamin D alone or in combination with calcium in RCTs was analysed by Yao et al. (2019). Our systematic review differs from other reviews and meta‐analysis in the fact that the target population is men and women over 65 years residing in community and LTC settings with or without risk of hip fracture. In this review, the incidence of hip fracture in people over ≥65 years is studied as a primary outcome. Besides this, incidence of non‐vertebral fracture in older people, the incidence of hip fracture in older women and the impact of vitamin D3 plus calcium supplementation on femoral neck bone mineral density are assessed as secondary outcomes. Furthermore, the efficacy of administering various doses of vitamin D3 plus calcium is estimated as a subgroup analysis. Oral supplementation is cost effective, safe and easy to implement among older people compared to parenteral routes (e.g. Annual injections of vitamin D) (O'Brien, 1998). Hence, this study aimed to assess the impact of daily oral supplementation of vitamin D3 plus calcium on the incidence of hip fracture in men and women over 65 years.

## 2 METHODS

### 2.1 Inclusion/exclusion criteria

In this review, the target population was adults over 65 years, both men and women residing in community and long‐term care settings with or without risk of hip fracture with no terminal illness or cognitive impairment. RCTs that used daily oral supplementation of vitamin D3, and calcium were included in this review. No restriction was imposed on daily dose of vitamin D3/calcium. No limits were set on the year of publication. Only studies published in English were included in this review. Also, RCTs that reported incidence of hip fracture as their primary or secondary outcome were included in this review. Ergocalciferol studies, injectable vitamin D, weekly, fortnightly, monthly or yearly supplementation of vitamin D, calcium only studies, vitamin D only studies, studies on vitamin D and calcium plus another component such as milk, hormone replacement therapy, exercise and risedronate were excluded from this review.

### 2.2 Primary/secondary outcome

The primary outcome assessed in this study is the incidence of hip fracture in people over 65 years. Hip fracture is a fracture of the femur above a point 5 cm below the distal part of the lesser trochanter (Gillespie 2001). The incidence of non‐vertebral fracture, the incidence of hip fracture in women over 65 years and femoral neck bone mineral density are assessed as secondary outcomes. Beside the primary and secondary outcomes, subgroups by dosage are used to evaluate the efficacy of administering 800 IU of vitamin D3 plus 1200 mg calcium versus 800 IU vitamin D3 plus 1000 mg calcium in reducing the incidence of hip fracture and other non‐vertebral fractures.

### 2.3 Search strategy

Between October 2019 and January 2020, a systematic literature search was undertaken to ensure that all published data relating to the topic were identified. The following databases were searched: Cochrane Library, Embase, Medline, PubMed, Cumulative Index to Nursing and Allied Health Literature (CINAHL), Web of Science and Scopus.

Also, the reference list of the retrieved articles was scanned to identify more studies. Editorials, articles, letter from readers, etc., were also explored to complete the non‐indexed searching in the database. There was no limit set on the year of publication. Studies were selected from the date of inception of the database. Only studies published in English were considered. Unpublished articles were not included in this review. The last search was carried out on 17 January 2020. An initial scoping exercise was performed to identify relevant keywords. Subject headings and MeSH terms were then identified and combined using Boolean tools AND, and OR which are listed in Appendix A.

After searching the databases and other sources, the retrieved articles were screened based on the title and abstract. After removing duplicates and irrelevant studies, the remaining full‐text articles were reviewed for eligibility. Out of these full‐text articles, the RCTs that met the predefined inclusion exclusion criteria were included in this review. The screening and selection of the articles were carried out by the primary author and verified by second review author.

### 2.4 Data collection and analysis

Relevant data were extracted from the eligible studies using a data extraction table (Table 1). The extracted data were reviewed by the two co‐authors. Quality appraisal of the included studies was carried out using Cochrane Risk of Bias Tool (Higgins et al., 2011). The risk of bias tool incorporates six domains of bias namely selection bias, performance bias, detection bias, attrition bias, reporting bias and other bias. Bias is assessed as a judgement, that is high, low or unclear. Random sequence generation (the methods used by the researchers to randomly assign participants to intervention group and control group) and allocation concealment (the person who does randomise the patients is not aware of the next step of the treatment) are the techniques to prevent selection bias (Barcot et al., 2019). Reporting bias is selective/distorted reporting of results and available information (McGauran et al., 2010). Performance bias occurs when one group of participants receive more attention from the investigators (McCambridge et al., 2014). Blinding the outcome assessors reduces the risk of detection bias (Probst et al., 2016). Attrition bias occurs when the participants leave the study (Dumville et al., 2006). The quality of the included studies was verified by two authors.

**TABLE 1:** Data extraction table

Author & Year	No: of participants (% of women)	Age/ mean age	Country and study setting	Type of study	Medication in intervention group: oral supplementation of		Control group	Trial Duration	Hip fracture incidence	Non‐vertebral fracture	Femoral neck Bone mineral density	Risk of bias
					Vit D3 (IU)	Calcium (mg)						
Chapuy et al. (1992)	2790 (100)	69–106 years	France Nursing homes or apartment houses for elderly people	RCT	800	1200 (tricalcium phosphate)	Placebo	18 months	The total incidence of hip fracture is 80/1387 (Intervention group), 110/1403 (Control group). (*p* = 0.043)	Non vertebral fracture: 160/1387 (intervention group), 215/1403 (control group (*p* = 0.015)	Femoral BMD +2.9 + _6.4 (intervention group), +1.8 + _9.4 (control group) (*p* = 0.036)	Randomly assigned to vitamin D ‐ calcium group or the placebo group in groups of 4., Placebo controlled, ITT analysis
Chapuy et al. (1994)	2303 (100)	84 (mean age)	France, Nursing homes or apartment houses for elderly people	RCT	800	1200 (tricalcium phosphate)	Placebo	36 months	Hip fracture: 138/1176 (intervention group), 184/ 1127 (control group) (OR = 0.70; 95% CI, 0.62,0.78)	Non vertebral fracture: 301/1176 (Intervention group), 368/1127 (Control group). (OR 0.70; 95% CI, 0.51,0.91)	NA	Unclear Randomization method, Placebo controlled, ITT analysis
Dawson‐Hughes et al. (1997)	389 (55)	65+	United States Community Dwelling	Double blinded, placebo‐ controlled trial	700	500 (Calcium citrate malate)	Placebo	36 months	Hip fracture: 0/187 (intervention group), 1/202 (Control group)	Non vertebral fracture: 11/187 (intervention group), 26/202 (control group) (*p* = 0.03).	Femoral neck BMD: +0.18 + _1.90 (intervention group), −0.22 + _ 3.65 (control group), (*p* = 0.02).	Unclear randomization, Double blinded throughout treatment period, Placebo controlled, ITT analysis.
Chapuy et al. (2002)	583 (100)	Mean age 85 years	France Apartment Houses for the elderly.	RCT	800	1200 (tricalcium phosphate)	Placebo	24 months	Hip fracture: 27/393 (Intervention Group), 21/190 (Control group) (*p* = 0.07)	Non vertebral fracture: 69/393 (intervention group), 34/190 (control group)	Femoral neck BMD ‐1.2 + _6.4 (intervention group). ‐4.5 + _ 7.1 (control group)	Unclear randomization method, Double blinded throughout treatment period, Placebo controlled, ITT analysis
Porthouse et al. (2005)	3314 (100)	≥ 70 years	England Practice nurse led clinics in primary care.	Pragmatic open RCT	800	1000 (calcium carbonate)	Nil	18–42 months	Hip fracture 8/1321 (Intervention group), 17/1993 (Control group). OR 0.75; 95% CI, 0.31,1.78. *p* = 0.51	Non vertebral fracture: 58/1321 (intervention group), 91/1993 control group). OR 1.01; 95% CI, 0.71, 1.43)	NA	Computer based randomization, Pragmatic, open controlled trial, No medication in control group, ITT analysis
RECORD Trial Group (2005)	2638 (85)	70 years or older	UK 21 hospitals in the UK	RCT	800	1000 (calcium carbonate)	Placebo	24–62 months	Hip fracture 46/1306 (Intervention group), 41/1332 (control group)	Non vertebral fracture: 165/ 1306 (intervention group), 178/1332 (control group).	NA	Computer based randomization, Double blinded throughout treatment period, Placebo controlled, Masking of treatment allocation stated, ITT analysis
Salovaara et al. (2010)	375 (100)	65–71	Finland Bone and cartilage Research unit of the clinical research centre of the University of Kuopio, Finland	RCT	800	1000 (calcium carbonate)	Nil	52 months	Hip fracture 4/290 (intervention group), 2/313 (control group) Hazard Ratio 2.19 (0.40–12.00)	Non vertebral fracture: 77/290 (intervention group), 82/313 (control group) HR 0.89 (0.65–1.22)	NA	Randomization done by an independent statistician using Windows 11.0, Participants in the control group did not receive any intervention or placebo, ITT analysis

Each of the included studies evaluated the impact of daily oral supplementation of vitamin D3 (cholecalciferol) plus calcium on the incidence of hip fracture in older people across several different settings. A meta‐analysis using Revman 5.3 was used to analyse the data. The odds ratio (OR) of the incidence of hip fracture in men and women above 65 years is the primary measure of treatment effect. Odds ratio is the ratio of number of events to the number of non‐events. Mantel–Haenszel odds ratios were used in this study as the data were dichotomous (Cochrane handbook). *I* ^*2*^ was used to assess the statistical heterogeneity of the studies (Higgins et al., 2003). Fixed effect was used in this review because the included studies had similar design, intervention, patient population and outcome measures (Nikolakopoulou et al., 2014).

## 3 RESULTS

### 3.1 Literature search result

**FIGURE 1:**
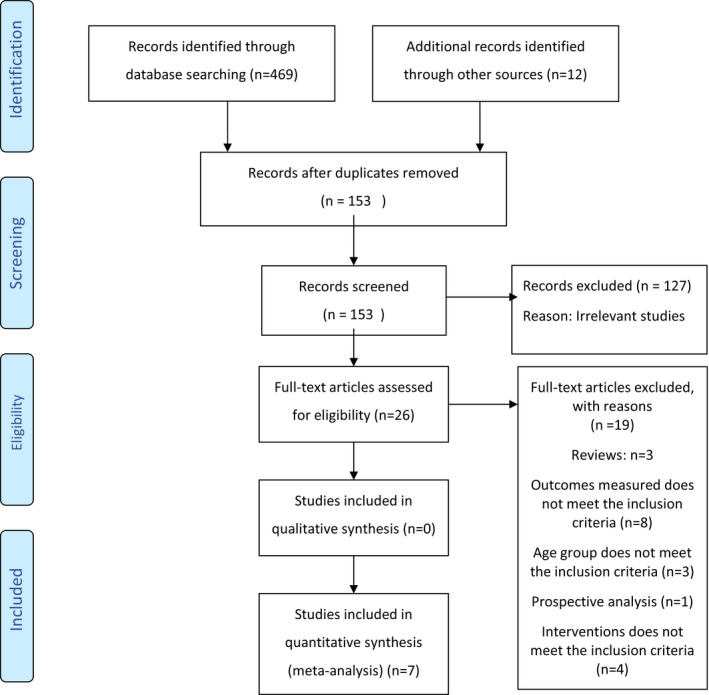
Prisma flow chart

A total of 481 articles were identified through database searching and other sources. Initial screening of the articles was based on title and abstract. After removing duplicates (*n* = 153) and irrelevant studies (*n* = 127), 26 full‐text articles were reviewed for eligibility. Out of the 26 full‐text articles, 19 were excluded as it did not meet the inclusion criteria. Seven RCTs that met the predefined inclusion/exclusion criteria were included in this review. Table 2 summarises all the excluded studies and reasons for exclusion, and the PRISMA Flowchart (Figure 1) outlines the study selection process.

**TABLE 2:** List of excluded studies with reason for exclusion

Study	Reason for exclusion
Meunier et al. (1994)	This is a review and hence does not meet the inclusion criteria
Kärkkäinen et al. (2010)	Outcome measured was the incidence of falls. So, it does not meet the inclusion criteria
Grados et al. (2003)	This study was excluded as the outcome measured does not fit the inclusion criteria
Pfeifer et al. (2009)	Outcome measured was the incidence of falls. So, it does not meet the inclusion criteria
Pfeifer et al. (2002)	Outcome measured was secondary hyperparathyroidism and body sway. This does not meet the inclusion criteria
Rees &Howe (2000)	This study compared the acceptability of CD3 forte and Ad Cal D3. Hence, it does not meet the inclusion criteria
Den Uyl et al. (2010)	This study does not fit in the inclusion criteria as it investigated the preference for and acceptability of chewable tablet containing calcium and Vitamin D compared to that of a sachet containing calcium and vitamin D3
Aloia et al. (2013)	This study was excluded as the outcome measured was the influence of calcium and vitamin D supplementation on parathyroid hormone and bone turn over. This does not meet the inclusion criteria
Bolland et al. (2015)	This is a systematic review. So, it does not meet the inclusion criteria
Bischoff‐Ferrari et al. (2005)	This study was excluded as it is a review
Prentice et al. (2013)	This study was excluded as the age group of the participants were 50–79 which does not meet the inclusion criteria
López‐Torres Hidalgo (2011)	Outcome measured does not meet the inclusion criteria
Jackson et al. (2006)	Age group of the participants were 50–79 which does not meet the inclusion criteria
Sato et al. (2005)	The intervention included administration of risedronate sodium plus calcium and vitamin D. This does not meet the inclusion criteria
Kärkkäinen et al. (2010)	The outcome measured does not fit in inclusion criteria
Feskanich et al. (2003)	This study is a prospective analysis which studied the impact of calcium, vitamin D, plus milk on the incidence of hip fracture. So, this study does not meet the inclusion criteria
Di Daniele N (2004)	This study was excluded as the age group of the participants (over 45 years) does not meet the inclusion criteria
Xia et al. (2009)	Both groups were treated with calcium and vitamin D
Harwood et al. (2004)	Incidence of fracture is not measured in the study

### 3.2 Geographical location

Of the seven RCTs included, three studies were conducted in France (Chapuy et al., 1992, 1994, 2002), two were conducted in the UK (Porthouse et al., 2005 & RECORD trial group 2005), one study in the United States (Dawson‐Hughes et al., 1997) and one in Finland (Salovaara et al., 2010).

### 3.3 Participant and sample size

The seven studies analysed a total of 12,620 older adults, of which 6060 (48%) were assigned to the intervention group and 6560 (51.9%) to the control group. There were 9593 (76%) women (intervention group = 4567 (47.6%) and control group = 5026 (52.3%)). The total number of men in the intervention group and control group is not mentioned in the RECORD trial study (2005). Hence not able to give an overall picture of the male participants included in this review. All participants were >65 years of age. All participants were either community dwelling or residents in long‐term care settings with or without risk of hip fractures.

### 3.4 Study design and settings

Out of the seven included RCTs, four studies were conducted among community‐dwelling older adults (Dawson‐Hughes et al., 1997, Porthouse et al., 2005, RECORD trial group 2005 and Salovaara et al., 2010) and three studies were conducted among ambulatory older women living in nursing homes or apartment houses for older people Chapuy et al. (1992, 1994, 2002).

### 3.5 Interventions and follow‐up

Three studies used 800 IU of vitamin D3 and 1200 mg of tricalcium phosphate for the intervention group and placebo for the control group (Chapuy et al., 1992 (treatment group = 1387 and control group = 1403), Chapuy et al., 1994 (treatment group = 1176 and control group = 1127) and Chapuy et al., 2002 (treatment group =393 and control group = 190)). One study used 700 IU of vitamin D3 plus 500 mg of calcium in the form of calcium citrate malate daily for the intervention group (*n* = 187) and placebo for the control group (*n* = 202) (Dawson‐Hughes et al., 1997). Three studies used 800 IU vitamin D3 and 1000 mg calcium in the form of calcium carbonate for the intervention group (Porthouse et al., 2005, intervention group 1321), (RECORD Trial Group, 2005, intervention group = 1306) and (Salovaara et al., 2010, intervention group = 290). In RECORD trial group study (2005), the control group (*n* = 1332) received placebo, whereas in the other two studies(Porthouse et al., 2005, control group = 1993, and Salovaara et al., 2010, control group = 313), the control group did not receive any treatment. The interventions used in the included studies are given in Table 3. The duration of the studies varied from 18 months to 62 months.

**TABLE 3:** Interventions used in the included studies

Author & year	Medications in intervention group: Oral supplementation of	
	Vitamin D3 (IU)	Calcium (mg)
Chapuy et al. (1992)	800	1200 mg (Tricalcium Phosphate)
Chapuy et al. (1994)	800	1200 mg (Tricalcium Phosphate
Dawson Hughes et al. (1997)	700	500 (Calcium citrate malate)
Chapuy et al. (2002)	800	1200 mg (Tricalcium Phosphate
Porthouse et al. (2005)	800	1000 mg (Calcium carbonate)
RECORD Trial Group (2005)	800	1000 mg (Calcium Carbonate)
Salovaara et al. (2010)	800	1000 mg (Calcium Carbonate)

### 3.6 Risk of bias in the study

Four studies had reasonable random sequence generation (Chapuy et al., 1992; Porthouse et al., 2005; RECORD Trial Group, 2005 and Salovaara et al., 2010). High risk for selection bias was noted in the study of Chapuy et al. (1994). Likewise, unclear risk of random sequence generation was identified in two studies (Chapuy et al., 2002; Dawson‐Hughes et al., 1997). Allocation concealment was clearly stated in only one study (RECORD trial group, 2005). In rest of the studies, the allocation concealment was either not described or not described in sufficient detail to allow a definite judgement making it unclear risk.

Low risk of performance bias was noted in six studies (Chapuy et al., 1992, 1994; Dawson‐Hughes et al., 1997; Porthouse et al., 2005; RECORD trial group, 2005; Salovaara et al., 2010). Detection bias risk was low in five studies (Chapuy et al., 2002; Dawson‐Hughes et al., 1997; Porthouse et al., 2005; RECORD trial group, 2005; Salovaara et al., 2010) and unclear in two studies (Chapuy et al., 1992, 1994). High risk for attrition bias was noted in the study of Porthouse et al. (2005). Low risk for attrition bias was identified in four studies (Chapuy et al., 1992, 2002; Dawson‐Hughes et al., 1997 and RECORD trial group, 2005). Attrition bias remains unclear in the study of Chapuy et al., 1994 and Salovaara et al., 2010).

**FIGURE 2:**
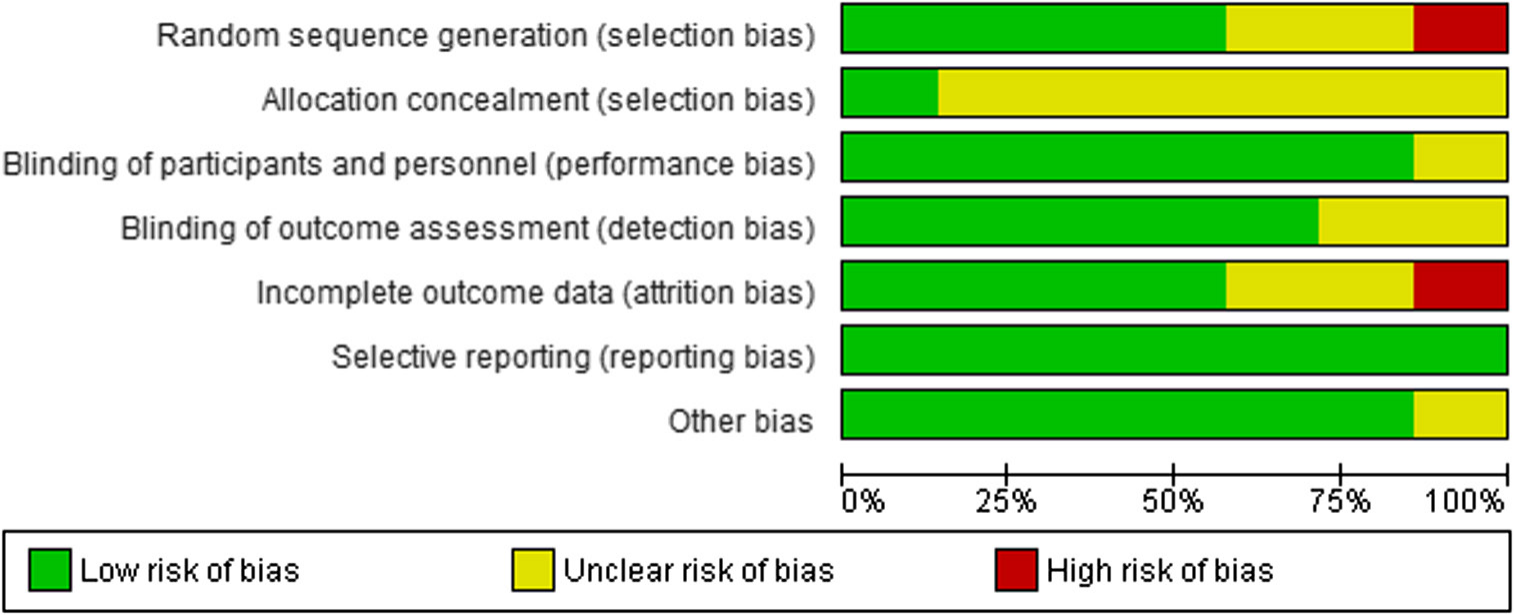
Risk of bias graph

**FIGURE 3:**
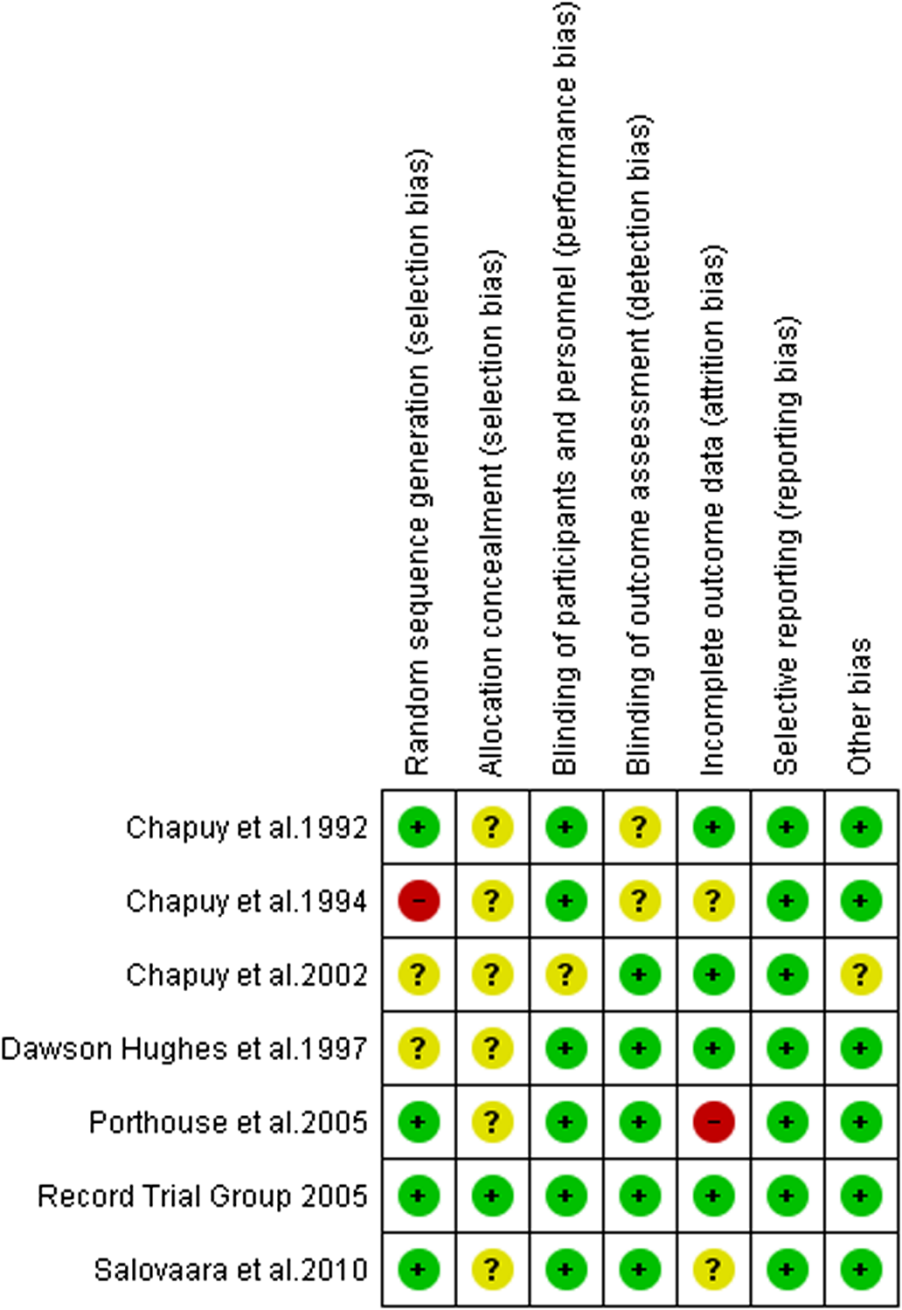
Risk of bias summary

Reporting bias risk was low in all the seven studies. Unclear risk of other sources of bias was identified in the study of Chapuy et al. (2002). Six studies appeared to be free from other sources of bias. Out of the seven studies, one study (RECORD trial group, 2005) showed low risk in all the six domains. Item level appraisal score for the included studies is included as a supplementary document in this manuscript. Risk of bias graph is included as Figure 2 and Risk of bias summary is included as Figure 3.

#### 3.6.1 Hip fracture and vitamin D3 & calcium supplementation

Chapuy et al. (1992) reported that the number of hip fractures was 43 per cent lower (*p* = .043) among the women treated with vitamin D3 and calcium than among those who received placebo. Chapuy et al. (1994) described that administering vitamin D3 and calcium reduces the risk of hip fracture (OR = 0.70; 95% CI: 0.62, 0.78). The study conducted by Chapuy et al. (2002) identified that the risk of hip fracture was higher in the placebo group compared to vitamin D3 and calcium group (*p* = .07). During the study, 27 of 393 women (6.9%) treated with calcium and vitamin D3 and 21 of 190 (11.1%) in the placebo group suffered from a hip fracture. In the study of Dawson‐Hughes et al. (1997), one hip fracture was reported in the placebo group (*n* = 202) and no hip fracture was reported in the vitamin D3 and calcium group (*n* = 187). Porthouse et al. (2005) reported that in their study, the OR for hip fracture was 0.75; 95% CI: 0.31, 1.78. *p* = .51. In the study of Salovaara et al., the hazard ratio (HR) associated with hip fracture was 2.19 (0.40–12.00), The study of Porthouse et al. (2005), RECORD trial group (, 2005) and Salovaara et al. (2010) concluded that administering vitamin D and calcium did not reduce the fracture risk in older adults.

### 3.7 Result of meta‐analysis

**FIGURE 4:**
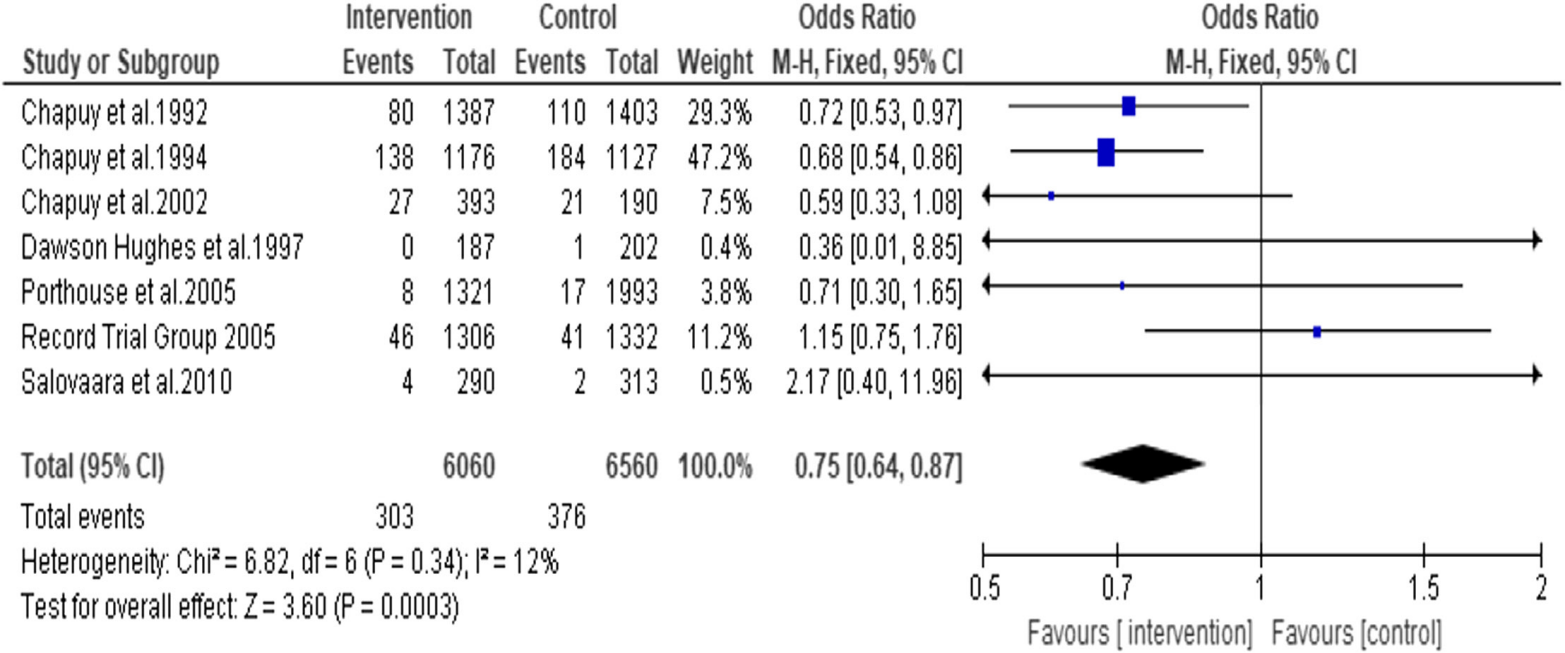
Forest plot on the incidence of hip fracture in people over 65 years

The meta‐analysis of the seven included RCTs showed OR of 0.75; 95% CI: 0.64, 0.87; *p* = .0003 indicating that combined vitamin D3 and calcium supplementation reduces hip fracture in older people by approximately 25% compared with placebo or no supplementation (Figure 4). No statistical heterogeneity was noted in the studies. I^2^ = 12%.

**FIGURE 5:**
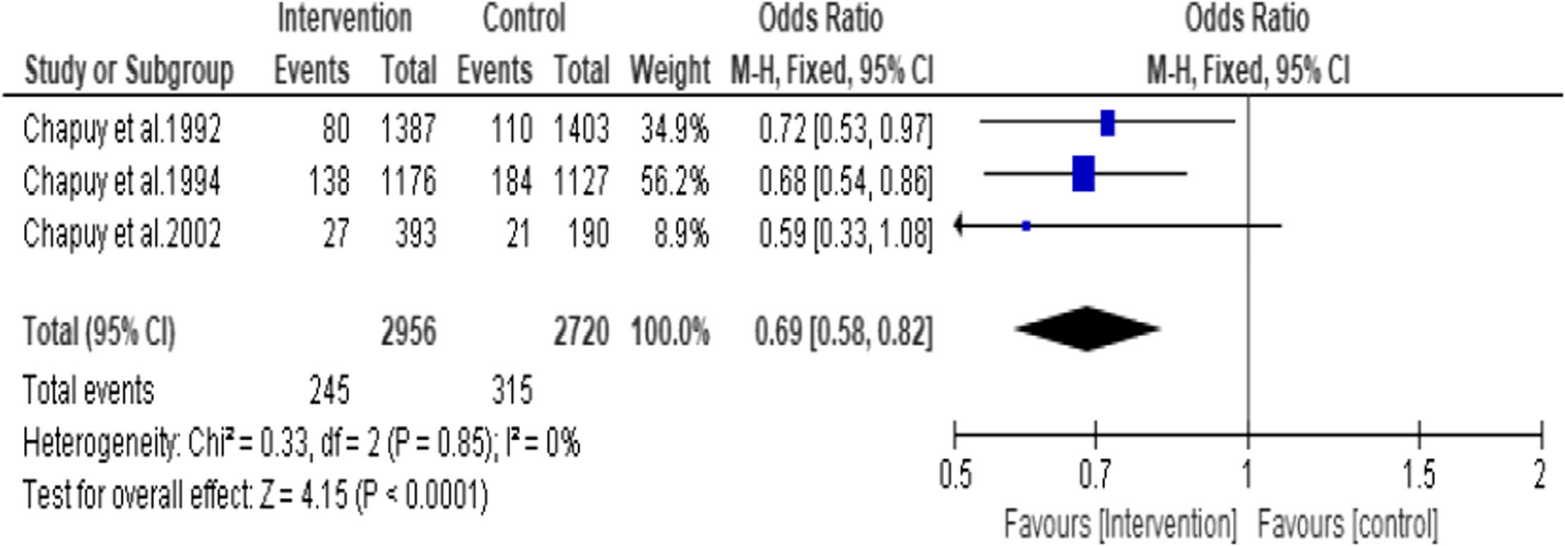
Subgroup1: Incidence of hip fracture in the studies that used 800 IU of Vitamin D3 plus 1200 mg of Calcium

A subgroup analysis was undertaken including three studies (Chapuy et al., 1992, 1994, 2002) that used 800 IU of vitamin D3 and 1200 mg of calcium as tricalcium phosphate showed a statistically significant reduction in the hip fracture in the intervention group by 31% (OR = 0.69, CI 95%: 0.58, 0.82; *p* < .0001) (Figure 5). A total of 5676 participants were included in this analysis (Intervention group *n* = 2956 and control group *n* = 2720). No statistical heterogeneity was noted among the studies. I^2^ = 0.

**FIGURE 6:**
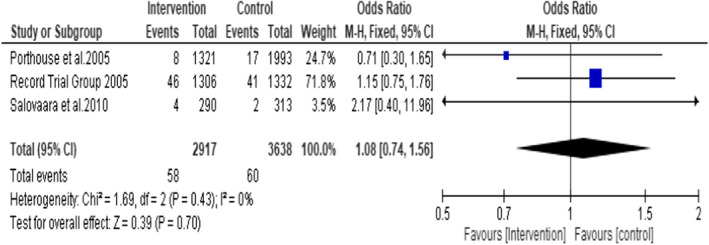
Subgroup 2: Incidence of hip fracture in the studies that used 800 IU of Vitamin D3 plus 1000 mg of Calcium

A subgroup analysis on the studies (Porthouse et al., 2005, RECORD trial group 2005 and Salovaara et al., 2010) (participants *n* = 6555, treatment group *n* = 2917 and control group *n* = 3638) that used 800 IU of vitamin D3 plus 1000 mg of calcium in the form of Calcium Carbonate did not favour the intervention group (OR = 1.08; 95% CI: 0.74, 1.56; *p* = .70) (Figure 6). No statistical heterogeneity was noted among the included studies. I^2^ = 0.

#### 3.7.1 Non‐vertebral fracture and vitamin D3 and calcium supplementation

The studies conducted by Chapuy et al. (1992, 1994 and 2002) showed a significant reduction in the total number of non‐vertebral fractures among women treated with vitamin D3 and calcium than among those who received placebo. The total number of non‐vertebral fractures in the intervention group was reduced by 32% (*p* = .015) and 30%(OR 0.70; 95% CI, 0.51, 0.91), respectively, in the studies of Chapuy et al., 1992 and (Chapuy et al., 1994) compared to the placebo group. Chapuy et al. (2002) acknowledged that 17.9%of the placebo group and 17.8% of the intervention group had at least one non‐vertebral fracture during the study period. Dawson‐Hughes et al. (1997) reported in their study that the RR of any first non‐vertebral fracture in the vitamin D3 and calcium group compared to the placebo group was 0.4 (*p* = .03). In the study of Porthouse et al. (2005), the OR for non‐vertebral fracture was 1.01; 95% CI, 0.71, 1.43). The hazard ratio (HR) associated with non‐vertebral fracture was 0.89 (0.65–1.22) reported in the study of Salovaara et al. (2010). No significant reduction in the incidence of non‐vertebral fracture was identified in the studies of RECORD trial group (2005), Porthouse et al. (2005) and Salovaara et al. (2010).

**FIGURE 7:**
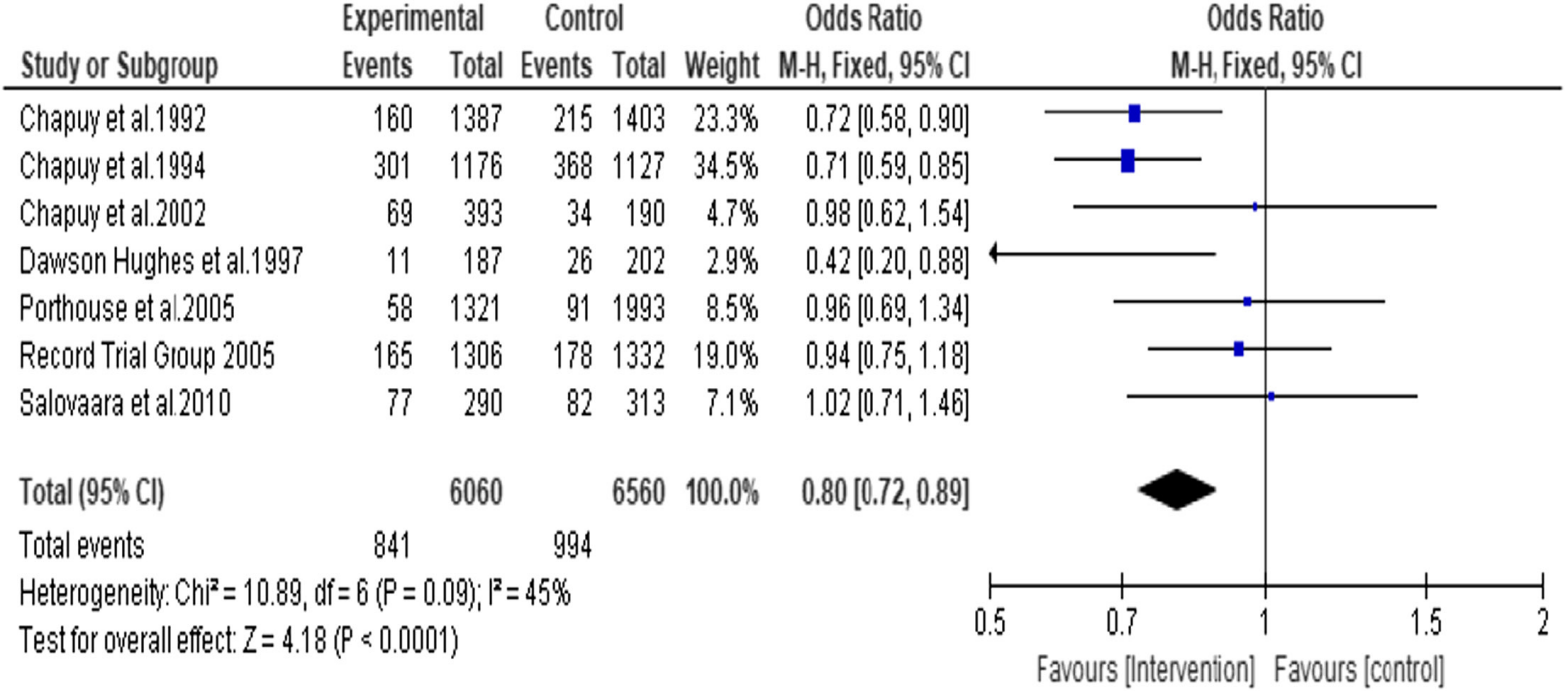
Incidence of all non‐vertebral fractures

A meta‐analysis of the seven included RCTs gave the odds ratio of 0.80 (95% CI: 0.72, 0.89; *p* < .0001), suggesting that daily oral supplementation of vitamin D3 and calcium reduces the incidence of all non‐vertebral fracture by 20% in intervention group compared to placebo or no supplementation group (Figure 7). No significant statistical heterogeneity was noted among the studies with I^2^ of 45%.

**FIGURE 8:**
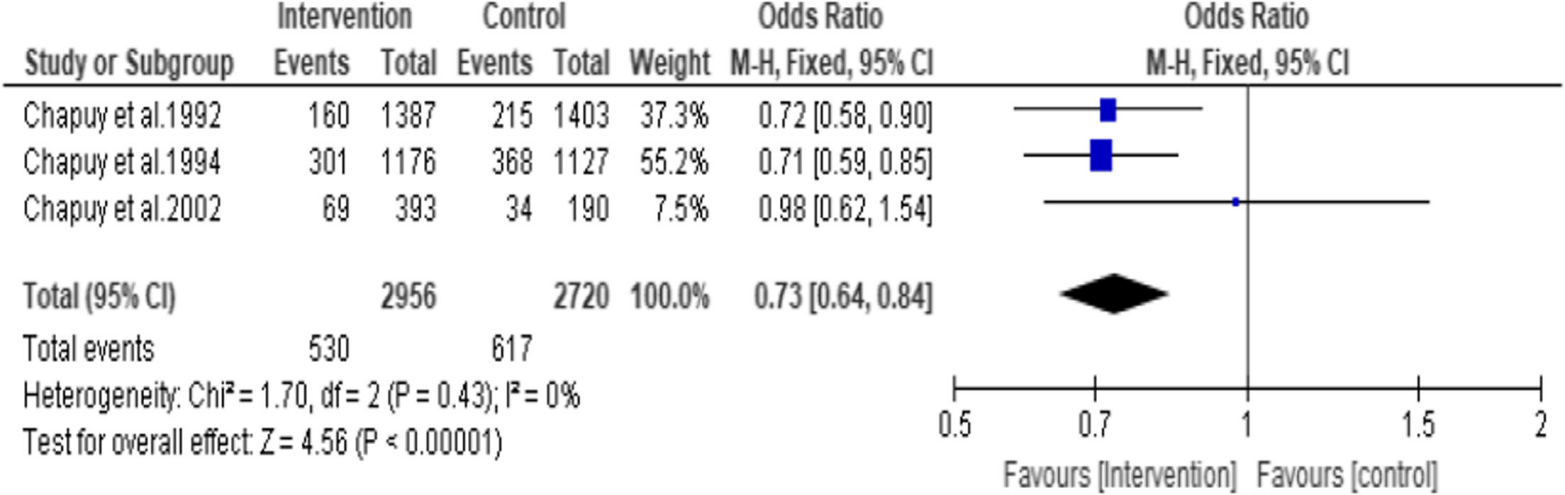
Subgroup 3: Incidence of non‐vertebral fractures in the studies that used 800 IU of Vitamin D3 plus 1200 mg Calcium

A subgroup analysis including three studies that administered 800 IU of vitamin D3 plus 1200 mg of calcium (Chapuy et al., 1992, 1994, 2002) showed a statistically significant reduction in the incidence of non‐vertebral fractures by 27% (OR = 0.73; 95% CI: 0.64, 0.84: *p* < .0001). A total of 5676 participants were included in this analysis (intervention group *n* = 2956 and control group *n* = 2720). (Figure 8). No statistical heterogeneity was noted among the studies I^2^ = 0.

**FIGURE 9:**
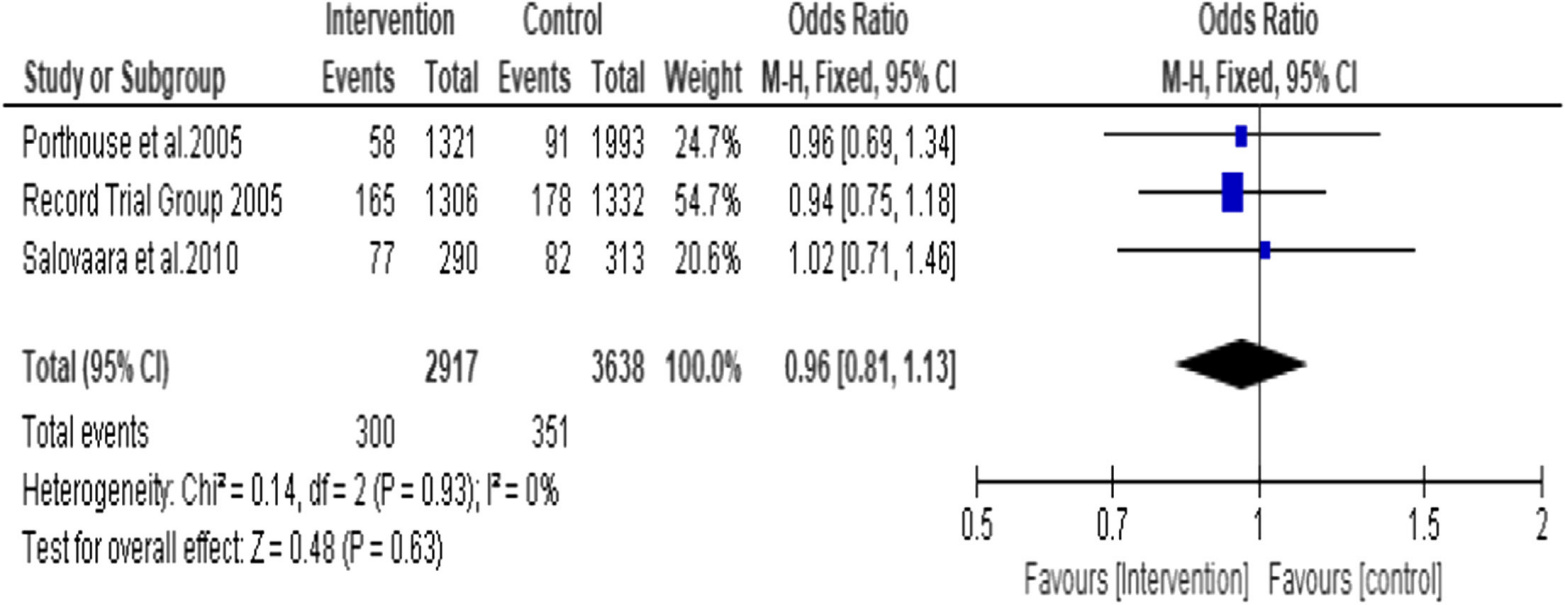
Subgroup 4: Incidence of non‐vertebral fractures in the studies that used 800 IU of Vitamin D3 plus 1000 mg Calcium

Another subgroup analysis including the three studies that used daily oral supplementation of 800 IU of vitamin D3 plus 1000 mg of calcium (Porthouse et al., 2005, Record trial group 2005 and Salovaara et al., 2010) showed no statistically significant effect (OR = 0.96 95% CI: 0.81, 1.13; *p* = .63). Participants *n* = 5555, intervention group *n* = 2917 and control group *n* = 3638 (Figure 9). No statistical heterogeneity was noted among the studies. I^2^ = 0.

#### 3.7.2 Hip fracture in women above 65 years

**FIGURE 10:**
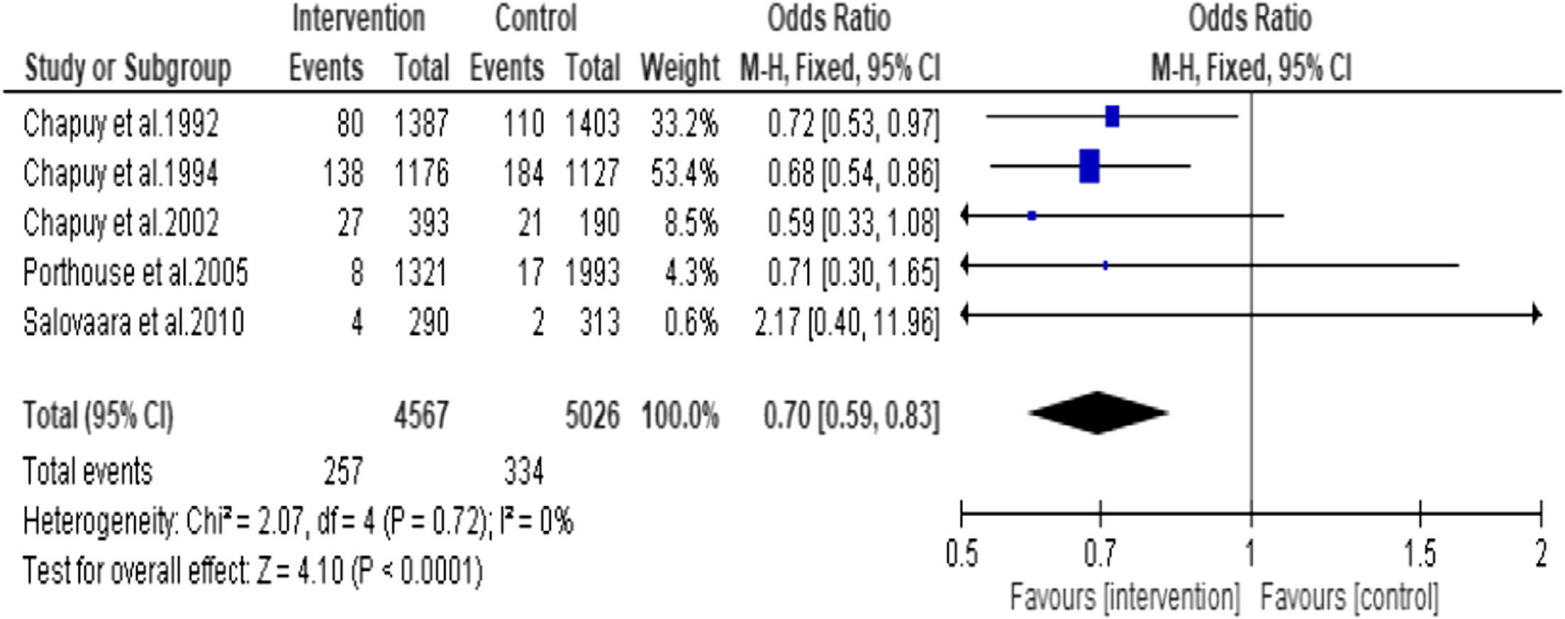
Forest plot on the incidence of hip fracture in older women

Five RCTs reported data on hip fracture in women above 65 years (Chapuy et al., 1992, 1994, 2002; Porthouse et al., 2005; Salovaara et al., 2010). Total participants *n* = 9593 (intervention group *n* = 4567 and control group *n* = 5026). A meta‐analysis of the five RCTs indicates an odds ratio of 0.70, (95% CI:0.59, 0.83; *p* < .0001) suggesting that daily oral supplementation of vitamin D3 and calcium reduced the incidence of hip fracture in older women by 30% compared to placebo or no supplementation (Figure 10). There was no statistically significant heterogeneity identified in these studies (I^2^ = 0%, *p* = 0.72).

#### 3.7.3 Association between vitamin D3 plus calcium supplement and femoral neck BMD

The femoral neck BMD was reported by three studies (Chapuy et al., 1992, 2002; Dawson‐Hughes et al., 1997). Chapuy et al. (1992) reported that the femoral neck BMD increased in the vitamin D and calcium group (*p* = 0.036). (Intervention group + 2.9 + _6.4 and control group + 1.8 + _9.4). Similarly, Chapuy et al. (2002) reported that the femoral neck BMD decreased in the placebo group (mean = −2.36%/year, SD = 4.92). Same was unchanged in the vitamin D3 and calcium group (mean = 0.29%/year, SD = 8.63, indicating that the treatment favours the intervention group. Dawson‐Hughes et al. (1997) reported that in their study, the mean (SD) changes in the femoral neck bone BMD (g/cm^2^) in the calcium–vitamin D and placebo groups were 0.504.80 and 0.705.03, respectively, (*p* = .02), indicating a significant increase in the femoral neck BMD.

**FIGURE 11:**
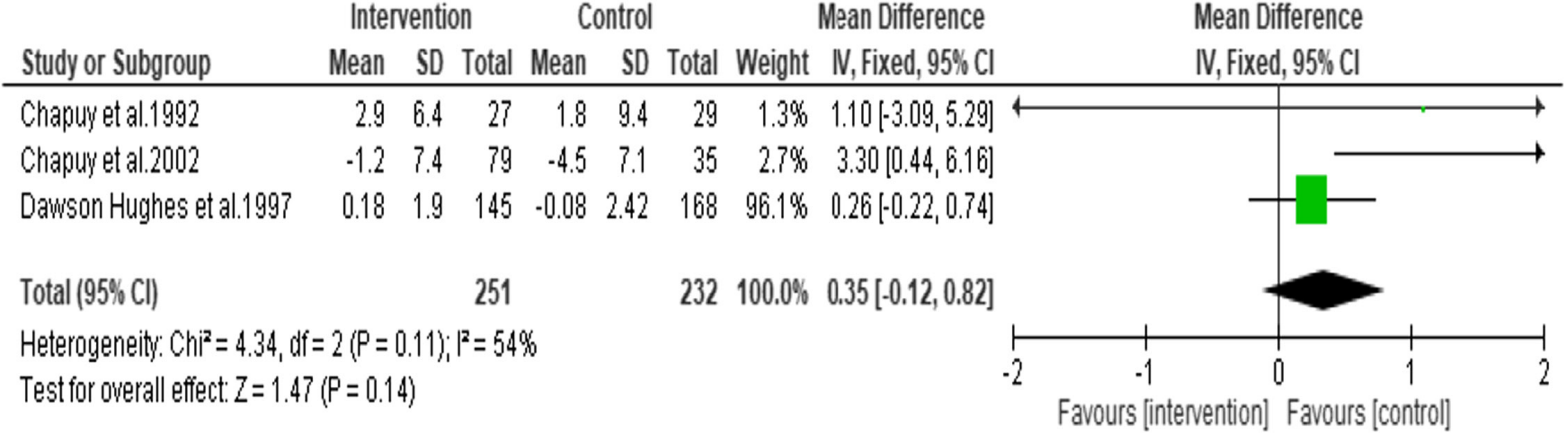
Forest plot on femoral neck bone mineral density

This was measured as a continuous outcome using fixed effect to combine the data. A meta‐analysis performed to assess the effects of daily oral supplementation of vitamin D3 plus calcium on femoral neck BMD showed no statistically significant difference (Mean difference = 1.21; 95% CI: −0.79, 3.20; *p* = .24) indicating that daily oral supplementation of vitamin D3 plus calcium does not favour increase in femoral neck BMD (Figure 11). A total of 483 subjects from three RCTs were included in this analysis. There was no statistically significant heterogeneity identified in these studies (I^2^ = 54% and *p* = .24).

## 4 DISCUSSION

This review attempted to recapitulate evidence from seven RCTs to identify the effectiveness of daily oral supplementation of vitamin D3 plus calcium on the incidence of hip fractures in older people. A total of 12,620 participants were included in this review. This review identified 25% reduction in hip fracture among older adults who received vitamin D3 and calcium supplementation. The subgroup analysis identified that daily oral supplementation of 800 IU of vitamin D3 plus 1200 mg of calcium is more effective in reducing hip fracture and non‐vertebral fracture compared to 800 IU vitamin D3 plus 1000 mg of calcium. There was a significant reduction of 20% in all non‐vertebral fractures and 30% reduction in hip fracture among older women. However, with regard to the femoral neck BMD, the supplementation with vitamin D3 and calcium did not favour the intervention group.

The findings of this review are similar to the findings of Avenell et al. (2014). Their review (Avenell et al., 2014) (nine trials, total participants = 49,853) identified that vitamin D plus calcium supplementation is effective in reducing hip fracture and non‐vertebral fracture in post‐menopausal women and older men. Their study was conducted among community, nursing home and hospital in patient population. Similarly, Boonen et al. (2007) reported that supplementation of vitamin D plus calcium showed an 18% reduction in hip fracture risk among post‐menopausal women and people ≥50 years of age. This study emphasised the benefits of using a combination of vitamin D and calcium in the prevention of hip fractures compared to the use of vitamin D alone. Tang et al. (2007) suggested that administering calcium alone or in combination with vitamin D is effective in preventing osteoporosis and reduces the risk of fracture. Tang et al. (2007) also acknowledged that the efficacy of same depends on compliance with the treatment. Similar findings were reported by Bergman et al. (2010).Reid and Bolland (2019) also stated that some meta‐analysis (Zhao et al., 2017) failed to show a consistent decrease in fracture risk despite combing vitamin D and calcium mainly due to low compliance. Reid and Bolland (2019)also pointed out that current evidence does not support the use of vitamin D and calcium supplements in healthy community‐dwelling adults due to the potential side effects. Heckman et al. (2002) described that daily supplementation of vitamin D and calcium reduces bone loss at the spine and femoral neck and reduces the risk of non‐vertebral fracture. The significance of combining vitamin D3 and calcium was highlighted in all these studies (Tang et al., 2007; Reid & Bolland, 2019; Heckman et al., 2002).

A limitation of this study is that the independent effects of calcium and vitamin D on hip fracture cannot be interpreted as the included studies used a combination of vitamin D3 and calcium supplementations. Depending on the baseline vitamin D level, the effectiveness of vitamin D plus calcium supplements may vary (Jackson et al., 2006). However, none of the studies reported on this. Another limitation is that only studies published in English were considered for the review and all the studies were performed in developed countries. This limits the generalisability of the findings to underdeveloped and developing countries.

## 5 CONCLUSION

A reduction in the incidence of hip fractures and all non‐vertebral fractures was noted among older adults who received vitamin D3 plus calcium supplementation. It was identified that 800 IU of vitamin D3 complemented with 1200 mg of calcium reduced hip fracture and non‐vertebral fracture significantly. At the same time, it should be noted that vitamin D3 plus calcium supplementation did not make any marked difference in femoral neck BMD. Future studies on potential adverse effects of vitamin D3 and calcium supplementation, studies to identify the efficacy of various doses and modes of vitamin D3 and calcium supplementation are warranted. It is advisable to review patient's calcium and vitamin D intake from all sources prior to commencing calcium and vitamin D supplements.

## 6 IMPLICATIONS FOR PRACTICE

Even though it is evident from the review that optimal daily intake of vitamin D3 plus calcium supplementation help in the prevention of fracture, it is only one essential element in fracture prevention. Also, people who are on dietary supplements should be compliant with same for better result. Efforts to prevent bone loss and osteoporosis should begin from an early age. It includes maintaining a healthy lifestyle, optimal intake of calcium and vitamin D3, proper nutrition, adequate exposure to sunlight, exercise etc. Proper education on healthy lifestyle, avoiding risk factors like smoking, caffeine, alcohol and awareness of bone health should continue throughout life with emphasis during menopause when increased bone loss is expected.

## CONFLICT OF INTEREST

There is no conflict of interest.

## ETHICS STATEMENT

Not required.

## Supporting information


Supplementary information files

## Data Availability

The authors declare that all relevant data supporting the findings of this study are available within the article and its (Supplementary information files).

## APPENDIX A

### A.1 Search Strategy

**CINAHL**

1. Vitamin D3 and calcium supplementation /or Caltrate /or Calcichew D3/or Vitamin D3 supplements/ or Calcium supplements/ or Cholecalciferol.
2. (vitamin d3* or cholecalciferol*).tw.
3. Or 1‐2
4. Hip fracture/ or neck of femur fracture /or Intertrochanteric fracture /or Intertrochanteric hip fracture
5. (neck of femur* fracture or nof* fracture).tw.
6. Or 4‐5
7. (bone mineral density or BMD*).tw.
8. Older adults /or Elderly /or Geriatric/ or Ageing
9. (ageing* or aging* or aged population).tw.
10. (older adults*or older people*).tw.
11. Or 8‐10
12. Randomised Controlled trial/ or Cluster randomised controlled trials.
13. (randomised controlled trial*or RCT*).tw
14. Or 12‐13
15. 3 and 6 and 7 and 11 and 14

(tw in this context is an instruction to search the title and abstract fields of the record) (line 5, 7,9 10 and 13 contains truncated words)

**Ovid MEDLINE(R) ALL <1946 to January 2020>**

1. (Vitamin D3 and Calcium).mp. [mp=title, abstract, original title, name of substance word, subject heading word, floating sub‐heading word, keyword heading word, organism supplementary concept word, protocol supplementary concept word, rare disease supplementary concept word, unique identifier, synonyms]: 3943
2. (vitamin d3* or cholecalciferol*).tw.: 13386
3. exp Vitamin D/ or exp Humans/ or exp Dietary Supplements/ or exp Calcium/ or exp Calcium, Dietary/ or Calcium Supplements.mp. or exp Aged/20715905
4. 1 and 2 and 3: 3288
5. Hip fracture.mp. or exp Hip Fractures/: 32052
6. exp Aged/ or exp Hip Fractures/ or exp Humans/ or exp Female/ or exp Femoral Fractures/ or exp Femoral Neck Fractures/ or neck of femur fracture.mp. or exp Male/: 22698697
7. exp Humans/ or exp Hip Fractures/ or exp Aged/ or Intertrochanteric hip fracture.mp.20508130
8. (neck of femur* fracture or nof* fracture).tw.: 218
9. 5 or 6 or 7 or 8: 22700685
10. 4 and 9 2834
11. (bone mineral density or BMD*).tw.: 54874
12. 10 and 11: 300
13. Older adults/ or Elderly/ or Geriatric/ or Ageing.mp. [mp=title, abstract, original title, name of substance word, subject heading word, floating sub‐heading word, keyword heading word, organism supplementary concept word, protocol supplementary concept word, rare disease supplementary concept word, unique identifier, synonyms] 3384744
14. (ageing* or aging* or aged population).tw.: 254465
15. older adults*or older people*.tw.0
16. 13 or 14 or 15 3523528
17. 12 and 16 135
18. Randomised Controlled trial.mp. or exp Randomized Controlled Trials as Topic/ or exp Clinical Trials as Topic/398259
19. randomised controlled trial*or RCT*.tw.: 0
20. 18 or 19: 398259
21. 17 and 20: 3

**Embase**

**Table:**

No. Query	Results
#7 #3 AND #4 AND #5 AND #6	0
#6 #1 AND #2	4
#5 ((randomised AND controlled AND trial OR cluster) AND randomised AND controlled AND trials. OR randomised) AND controlled AND trial*or AND rct*tw	0
#4 ((older AND adults OR elderly OR geriatric OR ageing OR ageing* OR aging* OR aged) AND population AND tw. OR older) AND adults*or AND older AND people* AND tw.	0
#3 (((vitamin AND d3 AND calcium AND supplementation OR caltrate OR calcichew) AND d3or AND vitamin AND d3 AND supplements OR calcium) AND supplements OR cholecalciferol.) AND bone AND mineral AND density OR bmd*	66,784
#2 ((((((vitamin AND d3 AND calcium AND supplementation OR caltrate OR calcichew) AND d3or AND vitamin AND d3 AND supplements OR calcium) AND supplements OR cholecalciferol.) AND hip AND fracture OR neck) AND of AND femur AND fracture OR intertrochanteric) AND fracture OR intertrochanteric) AND hip AND fracture	9,508
#1 ('calcium plus colecalciferol'/exp OR 'calcium plus colecalciferol') AND ('hip fracture'/exp OR 'hip fracture' OR (('hip'/exp OR hip) AND ('fracture'/exp OR fracture)))	44

**PubMed**

**Table:**

#6	Search: ((((Vitamin D3 and calcium supplementation /or Caltrate /or Calcichew D3/or Vitamin D3 supplements/ or Calcium supplements/ or Cholecalciferol.) AND (Hip fracture/ or neck of femur fracture /or Intertrochanteric fracture /or Intertrochanteric hip fracture)) AND (bone mineral density or BMD)) AND (Older adults /or Elderly /or Geriatric/ or Ageing/or Aging)) AND (Randomised Controlled trial/ or Cluster randomised controlled trials/or RCT) Filters: Free full text (((("cholecalciferol"[MeSH Terms] OR "cholecalciferol"[All Fields] OR ("vitamin"[All Fields] AND "D3"[All Fields]) OR "vitamin d3"[All Fields]) AND ("calcium"[MeSH Terms] OR "calcium"[All Fields] OR "calciums"[All Fields] OR "calcium s"[All Fields]) AND ("supplemental"[All Fields] OR "supplementating"[All Fields] OR "supplementation"[All Fields] OR "supplementation s"[All Fields] OR "supplementations"[All Fields] OR "supplemention"[All Fields])) OR ("calcium carbonate"[MeSH Terms] OR ("calcium"[All Fields] AND "carbonate"[All Fields]) OR "calcium carbonate"[All Fields] OR "caltrate"[All Fields]) OR ("Calcichew"[All Fields] AND "D3"[All Fields]) OR (("cholecalciferol"[MeSH Terms] OR "cholecalciferol"[All Fields] OR ("vitamin"[All Fields] AND "D3"[All Fields]) OR "vitamin d3"[All Fields]) AND ("dietary supplements"[MeSH Terms] OR ("dietary"[All Fields] AND "supplements"[All Fields]) OR "dietary supplements"[All Fields] OR "supplement"[All Fields] OR "supplement s"[All Fields] OR "supplemented"[All Fields] OR "supplementing"[All Fields] OR "supplements"[All Fields])) OR (("calcium"[MeSH Terms] OR "calcium"[All Fields] OR "calciums"[All Fields] OR "calcium s"[All Fields]) AND ("dietary supplements"[MeSH Terms] OR ("dietary"[All Fields] AND "supplements"[All Fields]) OR "dietary supplements"[All Fields] OR "supplement"[All Fields] OR "supplement s"[All Fields] OR "supplemented"[All Fields] OR "supplementing"[All Fields] OR "supplements"[All Fields])) OR ("cholecalciferol"[MeSH Terms] OR "cholecalciferol"[All Fields] OR "cholecalciferols"[All Fields] OR "colecalciferol"[All Fields])) AND ("hip fractures"[MeSH Terms] OR ("hip"[All Fields] AND "fractures"[All Fields]) OR "hip fractures"[All Fields] OR ("hip"[All Fields] AND "fracture"[All Fields]) OR "hip fracture"[All Fields] OR ("femoral neck fractures"[MeSH Terms] OR ("femoral"[All Fields] AND "neck"[All Fields] AND "fractures"[All Fields]) OR "femoral neck fractures"[All Fields] OR ("neck"[All Fields] AND "femur"[All Fields] AND "fracture"[All Fields]) OR "neck of femur fracture"[All Fields]) OR ("hip fractures"[MeSH Terms] OR ("hip"[All Fields] AND "fractures"[All Fields]) OR "hip fractures"[All Fields] OR ("Intertrochanteric"[All Fields] AND "fracture"[All Fields]) OR "intertrochanteric fracture"[All Fields]) OR ("Intertrochanteric"[All Fields] AND ("hip fractures"[MeSH Terms] OR ("hip"[All Fields] AND "fractures"[All Fields]) OR "hip fractures"[All Fields] OR ("hip"[All Fields] AND "fracture"[All Fields]) OR "hip fracture"[All Fields]))) AND ("bone density"[MeSH Terms] OR ("bone"[All Fields] AND "density"[All Fields]) OR "bone density"[All Fields] OR ("bone"[All Fields] AND "mineral"[All Fields] AND "density"[All Fields]) OR "bone mineral density"[All Fields] OR "BMD"[All Fields]) AND ("aged"[MeSH Terms] OR "aged"[All Fields] OR ("older"[All Fields] AND "adults"[All Fields]) OR "older adults"[All Fields] OR ("aged"[MeSH Terms] OR "aged"[All Fields] OR "elderly"[All Fields] OR "elderlies"[All Fields] OR "elderly s"[All Fields] OR "elderlys"[All Fields]) OR ("geriatric"[All Fields] OR "geriatrics"[MeSH Terms] OR "geriatrics"[All Fields]) OR ("aging"[MeSH Terms] OR "aging"[All Fields] OR "ageing"[All Fields]) OR ("aging"[MeSH Terms] OR "aging"[All Fields] OR "ageing"[All Fields])) AND ("randomized controlled trial"[Publication Type] OR "randomized controlled trials as topic"[MeSH Terms] OR "randomised controlled trial"[All Fields] OR "randomized controlled trial"[All Fields] OR (("cluster analysis"[MeSH Terms] OR ("cluster"[All Fields] AND "analysis"[All Fields]) OR "cluster analysis"[All Fields] OR "clustering"[All Fields] OR "clusterings"[All Fields] OR "cluster"[All Fields] OR "cluster s"[All Fields] OR "clustered"[All Fields] OR "clusterization"[All Fields] OR "clusters"[All Fields]) AND ("randomized controlled trial"[Publication Type] OR "randomized controlled trials as topic"[MeSH Terms] OR "randomised controlled trials"[All Fields] OR "randomized controlled trials"[All Fields])) OR "RCT"[All Fields])) AND (ffrft[Filter]) Translations Vitamin D3: "cholecalciferol"[MeSH Terms] OR "cholecalciferol"[All Fields] OR ("vitamin"[All Fields] AND "d3"[All Fields]) OR "vitamin d3"[All Fields] calcium: "calcium"[MeSH Terms] OR "calcium"[All Fields] OR "calciums"[All Fields] OR "calcium's"[All Fields] supplementation /: "supplemental"[All Fields] OR "supplementating"[All Fields] OR "supplementation"[All Fields] OR "supplementation's"[All Fields] OR "supplementations"[All Fields] OR "supplemention"[All Fields] Caltrate /: "calcium carbonate"[MeSH Terms] OR ("calcium"[All Fields] AND "carbonate"[All Fields]) OR "calcium carbonate"[All Fields] OR "caltrate"[All Fields] Vitamin D3: "cholecalciferol"[MeSH Terms] OR "cholecalciferol"[All Fields] OR ("vitamin"[All Fields] AND "d3"[All Fields]) OR "vitamin d3"[All Fields] supplements/: "dietary supplements"[MeSH Terms] OR ("dietary"[All Fields] AND "supplements"[All Fields]) OR "dietary supplements"[All Fields] OR "supplement"[All Fields] OR "supplement's"[All Fields] OR "supplemented"[All Fields] OR "supplementing"[All Fields] OR "supplements"[All Fields] Calcium: "calcium"[MeSH Terms] OR "calcium"[All Fields] OR "calciums"[All Fields] OR "calcium's"[All Fields] supplements/: "dietary supplements"[MeSH Terms] OR ("dietary"[All Fields] AND "supplements"[All Fields]) OR "dietary supplements"[All Fields] OR "supplement"[All Fields] OR "supplement's"[All Fields] OR "supplemented"[All Fields] OR "supplementing"[All Fields] OR "supplements"[All Fields] Cholecalciferol.: "cholecalciferol"[MeSH Terms] OR "cholecalciferol"[All Fields] OR "cholecalciferols"[All Fields] OR "colecalciferol"[All Fields] Hip fracture/: "hip fractures"[MeSH Terms] OR ("hip"[All Fields] AND "fractures"[All Fields]) OR "hip fractures"[All Fields] OR ("hip"[All Fields] AND "fracture"[All Fields]) OR "hip fracture"[All Fields]
	neck of femur fracture /: "femoral neck fractures"[MeSH Terms] OR ("femoral"[All Fields] AND "neck"[All Fields] AND "fractures"[All Fields]) OR "femoral neck fractures"[All Fields] OR ("neck"[All Fields] AND "femur"[All Fields] AND "fracture"[All Fields]) OR "neck of femur fracture"[All Fields] Intertrochanteric fracture /: "hip fractures"[MeSH Terms] OR ("hip"[All Fields] AND "fractures"[All Fields]) OR "hip fractures"[All Fields] OR ("intertrochanteric"[All Fields] AND "fracture"[All Fields]) OR "intertrochanteric fracture"[All Fields] hip fracture: "hip fractures"[MeSH Terms] OR ("hip"[All Fields] AND "fractures"[All Fields]) OR "hip fractures"[All Fields] OR ("hip"[All Fields] AND "fracture"[All Fields]) OR "hip fracture"[All Fields] bone mineral density: "bone density"[MeSH Terms] OR ("bone"[All Fields] AND "density"[All Fields]) OR "bone density"[All Fields] OR ("bone"[All Fields] AND "mineral"[All Fields] AND "density"[All Fields]) OR "bone mineral density"[All Fields] Older adults /: "aged"[MeSH Terms] OR "aged"[All Fields] OR ("older"[All Fields] AND "adults"[All Fields]) OR "older adults"[All Fields] Elderly /: "aged"[MeSH Terms] OR "aged"[All Fields] OR "elderly"[All Fields] OR "elderlies"[All Fields] OR "elderly's"[All Fields] OR "elderlys"[All Fields] Geriatric/: "geriatric"[All Fields] OR "geriatrics"[MeSH Terms] OR "geriatrics"[All Fields] Ageing/: "aging"[MeSH Terms] OR "aging"[All Fields] OR "ageing"[All Fields] Aging: "aging"[MeSH Terms] OR "aging"[All Fields] OR "ageing"[All Fields] Randomised Controlled trial/: "randomized controlled trial"[Publication Type].or. "randomized controlled trials as topic"[MeSH Terms].or. "randomised controlled trial"[All Fields] .or. "randomized controlled trial"[All Fields] Cluster: "cluster analysis"[MeSH Terms] OR ("cluster"[All Fields] AND "analysis"[All Fields]) OR "cluster analysis"[All Fields] OR "clustering"[All Fields] OR "clusterings"[All Fields] OR "cluster"[All Fields] OR "cluster's"[All Fields] OR "clustered"[All Fields] OR "clusterization"[All Fields] OR "clusters"[All Fields] randomised controlled trials/: "randomized controlled trial"[Publication Type].or. "randomized controlled trials as topic"[MeSH Terms] .or. "randomised controlled trials"[All Fields] .or. "randomized controlled trials"[All Fields] Warnings ((((Vitamin D3 and calcium supplementation /or Caltrate /or Calcichew D3/or Vitamin D3 supplements/ or Calcium supplements/ or Cholecalciferol.) AND (Hip fracture/ or neck of femur fracture /or Intertrochanteric fracture /or Intertrochanteric hip fracture)) AND (bone mineral density or BMD)) AND (Older adults /or Elderly /or Geriatric/ or Ageing/or Aging)) AND (Randomised Controlled trial/ or Cluster randomised controlled trials/or RCT) Stop word: and
#5	Search: Randomised Controlled trial/ or Cluster randomised controlled trials./or RCTs Filters: Free full text
#4	Search: Older adults /or Elderly /or Geriatric/ or Ageing/or aging Filters: Free full text
#3	Search: bone mineral density or BMD Filters: Free full text
#2	Search: Hip fracture/ or neck of femur fracture /or Intertrochanteric fracture /or Intertrochanteric hip fracture Filters: Free full text
#1	Search: Vitamin D3 and calcium supplementation /or Caltrate /or Calcichew D3/or Vitamin D3 supplements/ or Calcium supplements/ or Cholecalciferol. Filters: Free full text

**Web of Science**

1. Vitamin D3 plus calcium supplementation and hip fracture older adults (All Fields) 3
2. ALL=(Vitamin D3 and calcium supplementation or Caltrate or Calcichew D3or Vitamin D3 supplements or Calcium supplements or Cholecalciferol. 18,822
3. (ALL=(Vitamin D3 and calcium supplementation or Caltrate or Calcichew D3or Vitamin D3 supplements or Calcium supplements or Cholecalciferol.)) AND ALL=(Hip fracture or neck of femur fracture or Intertrochanteric fracture or Intertrochanteric hip fracture or NOF fracture) 754
4. ((ALL=(Vitamin D3 and calcium supplementation or Caltrate or Calcichew D3or Vitamin D3 supplements or Calcium supplements or Cholecalciferol.)) AND ALL=(Hip fracture or neck of femur fracture or Intertrochanteric fracture or Intertrochanteric hip fracture or NOF fracture)) AND ALL=(bone mineral density or BMD) 472
5. ALL=(Older adults or Elderly or Geriatric or Ageing) 4,071,227
6. #4 AND #5 348
7. ALL=(Randomised Controlled trial or Cluster randomised controlled trials or RCT) 491,219
8. #6 AND #7 126

**SCOPUS**

TITLE‐ABS‐KEY ( vitamin AND d3 AND plus AND calcium AND supplements AND hip AND fracture AND older AND adults )

( TITLE‐ABS‐KEY ( vitamin AND d3 AND plus AND calcium AND supplements AND hip AND fracture AND older AND adults ) AND TITLE‐ABS‐KEY ( vitamin

TITLE‐ABS‐KEY ( hip AND fracture/ OR neck AND of AND femur AND fracture AND /or AND intertrochanteric AND fracture AND /or AND

TITLE‐ABS‐KEY ( bone AND mineral AND density OR bmd )

TITLE‐ABS‐KEY ( older AND adults AND /or AND elderly AND /or AND geriatric/ OR ageing )

TITLE‐ABS‐KEY ( randomised AND controlled AND trial/ OR cluster AND randomised AND controlled AND trials/or AND rct )

( TITLE‐ABS‐KEY ( vitamin AND d3 AND plus AND calcium AND supplements AND hip AND fracture AND older AND adults ) AND TITLE‐ABS‐KEY ( ( bone AND

( TITLE‐ABS‐KEY ( vitamin AND d3 AND plus AND calcium AND supplements AND hip AND fracture AND older AND adults ) ) AND ( TITLE‐ABS‐KEY ( hip AND

( ( TITLE‐ABS‐KEY ( vitamin AND d3 AND plus AND calcium AND supplements AND hip AND fracture AND older AND adults ) AND TITLE‐ABS‐KEY ( ( bone
