# Supplementary material for: What is the impact of daily oral supplementation of vitamin D3 (cholecalciferol) plus calcium on the incidence of hip fracture in older people? A systematic review and meta‐analysis
Source: Int J Older People Nurs. 2022 Jul 17;18(1):e12492. doi: 10.1111/opn.12492 (PMC10078370; doi:10.1111/opn.12492)
Supplement: Supplementary file 1 — Supplementary information files [file OPN-18-0-s001.docx]

**Risk of Bias**

**Item level appraisal score for the included studies.**

**Chapuy et al. (1992)**

| **Bias** | **Author’s judgement** | **Support for judgement** |
| --- | --- | --- |
| Random sequence generation (Selection bias) | Low risk | “In order to minimize social, nutritional and lifestyle differences between the treatment groups, the women were randomly assigned to the vitamin D3-Calcium group or the placebo group in groups of four.” |
| Allocation concealment (selection bias) | Unclear risk | Method of concealment is not described. |
| Blinding of participants and personnel (performance bias) | Low risk | “The supplements were taken at lunch time in the presence of a nurse to ensure compliance.” It is unlikely that the blinding could have been broken. Treatment group and the placebo group participants received pills and suspension of identical appearance. |
| Blinding of outcome assessment (detection bias) | Unclear risk | Insufficient information to permit judgement of “low risk” or “high risk” |
| Incomplete outcome data (attrition bias) | Low risk | “The dropout rates during the study were similar in the two groups (death 16% in the vitamin D3 and calcium group and 17% in the placebo group: withdrawal for other reasons, 30% in the vitamin D3- calcium group and 29% in the placebo group.” |
| Selective reporting (reporting bias) | Low risk | All pre specified outcomes were reported. |
| Other bias | Low risk | The study appears to be free of other sources of bias. |

**Chapuy et al. (1994)**

| **Bias** | **Author’s judgement** | **Support for judgement** |
| --- | --- | --- |
| Random sequence generation (Selection bias) | High risk | How the participants were randomised is not mentioned in the study. |
| Allocation concealment (selection bias) | Unclear risk | Method of concealment is not described. |
| Blinding of participants and personnel (performance bias) | Low risk | Blinding of participants and key study personnel ensured, and unlikely that the blinding could have been broken. Both groups received pills of identical appearance. |
| Blinding of outcome assessment (detection bias) | Unclear risk | The study did not address this outcome. |
| Incomplete outcome data (attrition bias) | Unclear risk | The study did not address this outcome. |
| Selective reporting (reporting bias) | Low risk | The pre specified outcomes were reported. |
| Other bias | Low risk | The study appears to be free of other sources of bias. |

**Chapuy et al. (2002)**

| **Bias** | **Author’s judgement** | **Support for judgement** |
| --- | --- | --- |
| Random sequence generation (Selection bias) | Unclear risk | Insufficient information to permit judgement of “low risk” or “high risk” |
| Allocation concealment (selection bias) | Unclear risk | Method of concealment is not described. |
| Blinding of participants and personnel (performance bias) | Unclear risk | “The study was conducted using the double dummy method” |
| Blinding of outcome assessment (detection bias) | Low risk | Blinding of outcome assessment ensured, and unlikely that the blinding could have been broken. |
| Incomplete outcome data (attrition bias) | Low risk | Missing data is unlikely to affect the final outcome as the mean compliance was more than 95%. |
| Selective reporting (reporting bias) | Low risk | No study protocol is available, but it is clear that the published reports include all expected outcomes. |
| Other bias | Unclear risk | The study was sponsored. But whether they were involved in the study or not is mentioned. |

**Dawson Hughes et al. (1997)**

| **Bias** | **Author’s judgement** | **Support for judgement** |
| --- | --- | --- |
| Random sequence generation (Selection bias) | Unclear risk | “The subjects were randomly assigned to either the placebo or the calcium- vitamin D group with stratification according to sex, race and decade of age. |
| Allocation concealment (selection bias) | Unclear risk | Insufficient information to permit judgement of “low risk” or “high risk” |
| Blinding of participants and personnel (performance bias) | Low risk | Blinding of participants and key study personnel ensured and unlikely that the blinding could have been broken. |
| Blinding of outcome assessment (detection bias) | Low risk | “The principal investigator was unaware of the subject’s study-group assignments” |
| Incomplete outcome data (attrition bias) | Low risk | Losses to follow up were disclosed. 87%of the enrolled participants who remained in the two study groups were included in the analyses of subjects who completed the study according to the protocol. This is not expected to affect the result. |
| Selective reporting (reporting bias) | Low risk | All pre specified outcomes were reported. |
| Other bias | Low risk | The study appears to be free of other sources of bias. |

**Porthouse et al. (2005)**

| **Bias** | **Author’s judgement** | **Support for judgement** |
| --- | --- | --- |
| Random sequence generation (Selection bias) | Low risk | Eligible women were randomised by computer at the York Trials Units by an independent person with no knowledge of the participant’s characteristics. |
| Allocation concealment (selection bias) | Unclear risk | Method of concealment is described. |
| Blinding of participants and personnel (performance bias) | Low risk | Incomplete blinding, but the review author judge that the outcome is not likely to be influenced by lack of blinding. |
| Blinding of outcome assessment (detection bias) | Low risk | No blinding of outcome assessment, but the review author judge that the outcome is not likely to be influenced by lack of blinding. |
| Incomplete outcome data (attrition bias) | High risk | “Adherence rates were only a little more than 60% at 12 months. This may have attenuated any effect of treatment” |
| Selective reporting (reporting bias) | Low risk | The study protocol is available and all of the study’s pre specified outcomes that are of interest in the review have been reported in the pre specified way. |
| Other bias | Low risk | The study declares the source of funding and reported that none of the funders were involved in the design, analysis or writing up of the study. |

**Record Trial Group 2005**

| **Bias** | **Author’s judgement** | **Support for judgement** |
| --- | --- | --- |
| Random sequence generation (Selection bias) | Low risk | Randomisation was centralised, computer generated; stratified by centre; and minimised by age (younger than 80 years or 80 years and older), sex, time since fracture (previous three months or longer) and type of fracture(proximal, femur, distal forearm, clinical vertebral or other). |
| Allocation concealment (selection bias) | Low risk | Masking of treatment allocation stated. |
| Blinding of participants and personnel (performance bias) | Low risk | “The allocation remained concealed until the final analyses (other than for confidential reports to the data monitoring committee)”. Each tablet had matching placebos. All materials were delivered by post every 4 months. |
| Blinding of outcome assessment (detection bias) | Low risk | “All outcomes were reported or verified by people who were masked to the allocation scheme” |
| Incomplete outcome data (attrition bias) | Low risk | Missing data have been imputed using appropriate methods. |
| Selective reporting (reporting bias) | Low risk | The study protocol is available and all of the study’s pre specified outcomes that are of interest in the review have been reported in the pre specified way. |
| Other bias | Low risk | The study declares the source of funding. Also, it is declared that “ no funding source had any role in the collection, management, analysis or interpretation of the data; writing of the report or the decision to submit the paper for publication. |

**Salovaara et al. (2010)**

| **Bias** | **Author’s judgement** | **Support for judgement** |
| --- | --- | --- |
| Random sequence generation (Selection bias) | Low risk | “The 3432 enrolled subjects were randomized into intervention (n=1718) and control (n=1714) groups by an independent statistician based on simple randomization . Randomization was performed with SPSS for Windows 11statistical software without any blocking or stratification.” |
| Allocation concealment (selection bias) | Unclear risk | Method of concealment is not described. |
| Blinding of participants and personnel (performance bias) | Low risk | “The subjects were informed by letter to which group they were randomized”. But the review author judge that the outcome is not likely to be influenced by lack of blinding. |
| Blinding of outcome assessment (detection bias) | Low risk | No blinding of outcome assessment, but the review author judge that the outcome measurement is not likely to be influenced by lack of blinding. |
| Incomplete outcome data (attrition bias) | Unclear risk | Reasons for missing data is provided. Same is insufficient to permit judgement of “low risk’ or ‘high risk ‘ |
| Selective reporting (reporting bias) | Low risk | All pre-specified primary and secondary outcomes were reported. |
| Other bias | Low risk | The study declares the source of funding. “None of the funding sources had any role in the design and conduct of study, collection, management, analysis and interpretation of the data; or preparation, review or approval of the manuscript. |
